# YKL-40 as a new promising prognostic marker of severity in COVID infection

**DOI:** 10.1186/s13054-020-03383-7

**Published:** 2021-02-16

**Authors:** Lauranne Schoneveld, Aurélie Ladang, Monique Henket, Anne-Noëlle Frix, Etienne Cavalier, Julien Guiot, the COVID-19 clinical investigators of the CHU de Liège, L. Schoneveld, A. Ladang, M. Henket, A. Frix, E. Cavalier, J. Guiot, A. Ancion, J. Berg, O. Bonhomme, A. Bouquegneau, C. Bovy, S. Bruls, G. Darcis, J. O. Defraigne, A. Ghuysen, A. Gilbert, V. Heinen, B. Lambermont, R. Louis, O. Malaise, M. Martin, B. Misset, M. Moutschen, D. Nguyen Dang, J. Piazza, D. Szecel, J. Szecel, H. Van Cauwenberge, C. Von Frenckell, L. Vroonen

**Affiliations:** 1 Department of Clinical Chemistry, University of Liege, CHU de Liège, Domaine Universitaire du Sart-Tilman, B35, 4000 Liège, Belgium; 2 Department of Pneumology, CHU de Liège, Liège, Belgium

**Keywords:** COVID-19, SARS-CoV-2, YKL-40, Chitinase 3-like 1, Interstitial lung disease

The severe acute respiratory syndrome coronavirus 2 (SARS-CoV-2) is responsible for a disease named COVID-19, which may be associated with common symptoms or lead patients to intensive care unit (ICU) or death. The severity of the disease is mainly driven by diffuse interstitial lung diseases (ILD). YKL-40 has a promitogenic action on pulmonary fibroblasts, increases the activity of macrophages and is associated with inflammatory disorders, arteriosclerosis and endothelial dysfunction. In ILD, YKL-40 has been described to be associated with the severity of lung diseases and with the risk of death [1–6]. Yet, in COVID-19 infection, YKL-40 serum levels could therefore be of interest for diagnosis and prognosis since it is at the cross-link between vascular and epithelial lung damage, which are typical characteristics of COVID-19 infection. By closing the gap between those two pathological characteristics, we thought that YKL-40 could be of interest a specific biomarker of severe COVID-19 infection.

We thus retrospectively compared serum levels of YKL-40 in a cohort of 103 patients infected by SARS-CoV-2 hospitalized between March 1 and April 29, 2020, with a group of 58 appariated healthy subjects (HS), 26 patients suffering from chronic obstructive pulmonary disease (COPD) and 53 from non-COVID ILD. Measurement of YKL-40 was taken with the MicroVue™ YKL-40 enzyme immunoassay kit during the 3 first days of admission and retrospectively analyzed and correlated the results with clinical data [ICU admission, acute renal failure (ARF) or multiple organ failure (MOF)].

Median age of COVID-19 positive patients was 69 yo with a male predominancy (67%). A significant proportion of the cohort (*n* = 103) experienced ICU admission (30%), ARF (32%) and MOF (28%).

COVID-19 patients who were admitted in ICU had statistically higher CRP, creatinine, LDH and YKL-40 (*p* < 0.05) (Table 1). The lymphocyte count was not statistically lower (*p* = 0.059) and D-dimers were not higher (*p* = 0.1297) compared to the other group.

**Table 1:** Comparison between COVID-19 patients admitted to intensive care or not

Variables	ICU, No (*n* = 72)	ICU, Yes (*n* = 31)	*p* value
Age (year)	71 (58–82)	65 (59–69)	< 0.05
Gender M/F	44/28	22/9	NS
Height (cm)	169 (162–176)	175 (169–180)	< 0.05
Weight (kg)	71 (63–84)	96 (80–105)	< 0.0001
BMI (kg/m^2^)	25 (22–29)	31 (27–34)	< 0.0001
Abnormal lung lesions (%)	20 (10–35)	40 (30–50)	< 0.001
SpO2 (%)	93 (89–96)	88 (75–90)	< 0.0001
Death, No/Yes (%)	92.8/7.2	89.3/10.7	NS
Shock or organ failure, No/Yes (%)	91.3/8.7	26.7/73.3	< 0.0001
Cardiopathy, No/Yes (%)	85.5/14.5	75/25	NS
ARF, No/Yes (%)	79.7/20.3	40/60	< 0.001
CRF, No/Yes (%)	85.5/14.5	96.7/3.3	NS
Diabetes, No/Yes (%)	85.5/14.5	62.1/37.9	< 0.05
Red blood cells (× 10e6/µl)	4.29 ± 0.79	4.48 ± 0.85	NS
Hematocrit (%)	38 ± 7	39 ± 7	NS
Hemoglobin (g/dl)	13 ± 2	14 ± 2	NS
Globular volume (fl)	89 ± 8	89 ± 7	NS
Reticulocytes (%)	0.97 (0.72–1.15)	1 (0.57–1.04)	NS
Reticulocytes (× 10^3^/µl)	39 (29–50)	39 (24–51)	NS
Leucocytes (× 10e3/µl)	6.15 (4.63–8.03)	7.87 (4.91–13.54)	< 0.05
Blood neutrophils (%)	73 ± 12	78 ± 17	< 0.05
Blood lymphocytes (%)	15 (10–24)	10 (5–20)	< 0.05
Blood monocytes (%)	0.2 (0–0.9)	0 (0–0.1)	< 0.01
Blood eosinophils (%)	7.73 ± 4.36	5.63 ± 3.32	NS
Blood basophils (%)	0.2 (0.2–0.4)	0.2 (0.1–0.3)	NS
Blood neutrophils (10^3^/µl)	4.54 (3.07–6.4)	6.96 (3.5–11.73)	< 0.05
Blood lymphocytes (10^3^/µl)	0.96 (0.69–1.27)	0.73 (0.59–1.12)	NS
Blood monocytes (10^3^/µl)	0.42 (0.25–0.6)	0.37 (0.27–0.6)	NS
Blood eosinophils (10^3^/µl)	0.01 (0–0.05)	0 (0–0.01)	< 0.05
Blood basophils (10^3^/µl)	0.01 (0.01–0.02)	0.02 (0.01–0.03)	NS
Platelets (10^3^/µl)	195 (157–266)	189 (155–252)	NS
Quick time (%)	83 ± 22	83 ± 13	NS
Quick time (s)	13 (12–13)	13 (12–13)	NS
Fibrinogen (g/l)	5.24 ± 1.61	6.06 ± 2.01	NS
D-dimers (µg/L)	876 (517–1787)	1483 (586–2422)	NS
Erythrocytes sedimentation rate (mm/h)	123 (123–123)	48 (48–48)	NS
Iron (µmol/l)	4.27 (2.95–7.36)	4.61 (3.35–7.06)	NS
Ferritin (µg/l)	827 (499–1677)	1861 (889–4117)	< 0.05
Osmolality (mosm/kg)	290 ± 15	286 ± 11	NS
Sodium (mmol/l)	139 ± 5	136 ± 4	< 0.05
Chlorides (mmol/l)	102 ± 6	100 ± 5	NS
Potassium (mmol/L)	4.04 ± 0.44	4.16 ± 0.62	NS
Calcium (mmol/l)	2.23 ± 0.18	2.14 ± 0.21	NS
Phosphates (mg/l)	0.99 ± 0.2	1.15 ± 0.34	NS
Bicarbonates (mmol/l)	24 (21–26)	23 (19–26)	NS
Creatinine (mg/dl)	0.93 (0.8–1.31)	1.25 (0.88–1.6)	0.05
Urea (mg/dL)	41 (31–68)	53 (40–84)	< 0.05
GFR (MDRD) (ml/min/1.73m^2^)	71 ± 33	60 ± 30	NS
Total protein (g/l)	66 ± 8	66 ± 11	NS
Albumin (g/l)	37 ± 5	36 ± 4	NS
Uric acid (mg/dl)	61 ± 25	63 ± 25	NS
CRP (mg/l)	58 (26–144)	166 (105–265)	< 0.0001
Total bilirubin (mg/dl)	0.62 (0.44–0.82)	0.79 (0.53–1.02)	< 0.05
Conjugated bilirubin (mg/dl)	0.25 (0.18–0.34)	0.33 (0.25–0.5)	< 0.05
Alkaline phosphatase (U/l)	75 (59–90)	70 (57–95)	NS
GGT (U/l)	52 (30–111)	64 (29–133)	NS
ASAT (U/L)	35 (24–53)	60 (35–80)	< 0.001
ALAT (U/L)	27 (17–46)	36 (26–56)	< 0.05
LDH (U/l)	310 (244–441)	503 (411–703)	< 0.00001
Lipase (U/l)	32 (19–50)	38 (25–53)	NS
Creatine kinase (U/l)	136 (59–266)	229 (101–426)	0.07
YKL-40 (ng/ml)	186 (84–384)	241 (172–827)	< 0.05

When the data follow a normal distribution, the results are expressed as mean ± standard deviation, and otherwise, they are expressed as the median (IQR)

M, male; F, female; NS, nonsignificant

COVID-19 patients exhibited higher serum levels of YKL-40 than HS, COPD and ILD (*p* < 0.0001 for all groups) (Fig. 1). Median serum level of YKL-40 was 206 ng/ml (95–431) in the COVID-19 group, 46 ng/ml (34–67) in the HS subgroup, whereas they were of 60 ng/ml (41–73) in the COPD and 73 ng/ml (42–91) in the ILD groups, respectively.

**Fig. 1:**
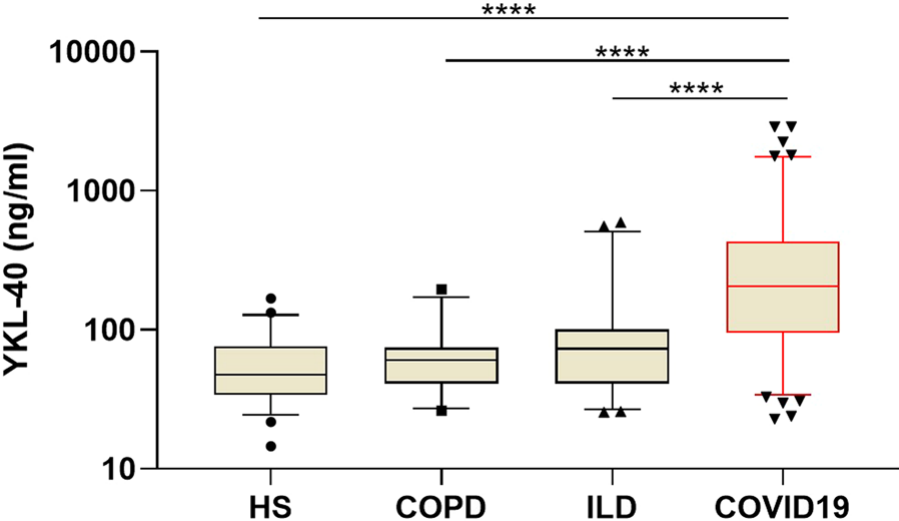
YKL-40 concentrations in Healthy Subjects (HS), Chronic Obstructive Pulmonary Disease (COPD), Interstitial Lung Diseases (ILD) and COVID-19 patients. A Kruskal–Wallis test and a post hoc Dunn test were used

Patients suffering from more severe diseases had significantly higher YKL-40 values than those who did not experience ICU admission, MOF or ARF (*p* < 0.05, *p* < 0.05, *p* < 0.001, respectively). Patients infected by COVID-19 suffering from prior chronic renal failure and chronic cardiopathy were exhibiting an increased serum level of YKL-40 (*p* < 0.0001 and *p* < 0.001, respectively). Death was not statistically correlated to levels of YKL-40 within the COVID-19 patient group (*p* = 0.12).

The area under the ROC curve (AUC) for the discrimination of patients admitted or not to the ICU in association with the levels of YKL-40, the age and the percentage of lesions visible on the thoracic scanner reached 0.78 (*p* < 0.0001). The positive predictive value was 70%, and the negative predictive value was 83%.

In conclusion, this study showed that firstly the COVID-19 patients had higher levels of YKL-40 compared to a control population (HS, COPD and ILD) and secondly that within the COVID-19 population YKL-40 was an indicator of the seriousness of infection since it is linked to complications such as admission to ICU, ARF or MOF. This marker could also be a predictive marker to anticipate management at the ICU and is useful for the prognosis of the onset of an ILD later. Future studies are also needed to assess the correlation between the levels of YKL-40 and pulmonary sequelae that patients with COVID-19 would develop.

## Data Availability

The datasets used and/or analyzed during the current study are available from the corresponding author on reasonable request.

## Abbreviations

ICU: Intensive care unit
ILD: Interstitial lung diseases
HS: Healthy subjects
COPD: Chronic obstructive pulmonary disease
MOF: Multiple organ failure
AUC: Area under the ROC curve
